# Systemic Therapeutic Options in Radioiodine-Refractory Differentiated Thyroid Cancer: Current Indications and Optimal Timing

**DOI:** 10.3390/cancers17111800

**Published:** 2025-05-28

**Authors:** Tamara Díaz Vico, Brezo Martínez-Amores Martínez, Luka Mihic Góngora, Paula Jiménez-Fonseca, Paloma Peinado Martín, Irene Grao Torrente, Alejandro García Muñoz-Nájar, Manuel Durán-Poveda

**Affiliations:** 1 Department of General Surgery, Hospital Universitario Rey Juan Carlos, 28933 Madrid, Spain; brezo.martinez@hospitalreyjuancarlos.es (B.M.-A.M.); irene.grao@quironsalud.es (I.G.T.); alejandro.garcia@hospitalreyjuancarlos.es (A.G.M.-N.); manuel.duran@hospitalreyjuancarlos.es (M.D.-P.); 2 Department of Health Sciences, Rey Juan Carlos University, 28933 Móstoles, Spain; 3 HM CIOCC MADRID (Centro Integral Oncológico Clara Campal), Hospital Universitario HM Sanchinarro, 28050 Madrid, Spain; lmihic@hmhospitales.com (L.M.G.); ppeinado@hmhospitales.com (P.P.M.); 4 Instituto de Investigación Sanitaria HM Hospitales, Facultad HM de Ciencias de la Salud, Universidad Camilo José Cela, 28692 Cañada, Spain; 5 Medical Oncology Department, Hospital Universitario Central de Asturias, ISPA, 33011 Oviedo, Spain; palucaji@hotmail.com

**Keywords:** thyroid cancer, multikinase inhibitors, radioiodine-refractory, tyrosine kinase, targeted therapy, adverse events

## Abstract

Thyroid cancer (TC) remains a prevalent malignancy, with over 820,000 global cases diagnosed in 2022. Differentiated thyroid carcinoma (DTC), primarily papillary and follicular types, accounts for most cases and has a favorable prognosis with total thyroidectomy and radioiodine (RAI) ablation. However, 5–15% of patients develop RAI-refractory (RAI-R) disease, leading to a significantly poorer outcome. For RAI-R patients, treatment decisions depend on disease progression. Active surveillance is suitable for indolent cases, while symptomatic or progressive disease requires systemic therapy. Multikinase inhibitors (MKIs) such as lenvatinib and sorafenib serve as first-line options, with cabozantinib recently approved for resistant cases. Additionally, novel targeted therapies, including RET and NTRK inhibitors, and immune checkpoint inhibitors, are under investigation, offering a personalized approach. A key challenge is determining the optimal timing for systemic therapy, balancing progression-free survival (PFS) benefits against MKI-related toxicities, which significantly impact quality of life (QoL). Molecular testing can identify actionable mutations, guiding therapy selection. Clinical guidelines (ATA, ESMO) recommend initiating treatment based on disease progression and patient condition, integrating strategies such as active surveillance, surgery, and radiotherapy when appropriate. Despite advances, systemic therapies carry significant adverse events (e.g., hypertension, fatigue, gastrointestinal toxicity), necessitating careful monitoring to prevent dose reductions or interruptions. A multidisciplinary approach is essential to optimize patient outcomes and maintain QoL. As targeted therapies continue to evolve, further research is needed to refine treatment sequencing and improve outcomes for RAI-R TC. This review synthesizes current evidence to guide clinical decision-making.

## Simple Summary

Thyroid cancer (TC) is a common malignancy, with over 820,000 cases diagnosed globally in 2022. Differentiated thyroid carcinoma (DTC), including papillary and follicular types, generally responds well to treatment, but 5–15% of patients develop radioiodine-refractory (RAI-R) disease, leading to a poorer prognosis. In such cases, treatment decisions depend on disease progression. While some patients can be monitored through active surveillance, those with symptomatic or progressive disease require systemic therapy. Multikinase inhibitors (MKIs) like lenvatinib and sorafenib are the first-line treatment for RAI-R DTC, with cabozantinib recently approved for resistant cases. Additionally, targeted therapies, such as RET and NTRK inhibitors, and immune checkpoint inhibitors, offer new treatment possibilities. However, systemic therapies have significant side effects, impacting quality of life (QoL). This review summarizes current evidence on treatment options, optimal timing, and sequencing, highlighting the need for further research to improve personalized care for RAI-R TC patients.

## 1. Introduction

Thyroid cancer (TC) is a relatively common malignancy, with an estimated 821,214 new cases worldwide in 2022, accounting for 4.1% of all cancer diagnoses [1]. In Spain, 5233 new cases and 314 annual deaths have been reported, which accounts for 0.27% of cancer-related mortality, according to the latest available data from the Global Cancer Observatory [1].

Differentiated thyroid carcinoma (DTC), encompassing predominantly papillary and follicular subtypes, comprises many TC cases [2,3]. Standard treatment typically includes total thyroidectomy followed by radioiodine (RAI) ablation to remove residual thyroid tissue and treat microscopic disease. This approach is generally effective, with a 10-year survival rate exceeding 90% in localized disease [4].

However, approximately 5–15% of patients with DTC develop RAI-refractory (RAI-R) disease [5,6]. In these patients, tumors no longer concentrate iodine, making RAI therapy ineffective and leading to a markedly poorer prognosis, with a median overall survival (OS) of 2.5 to 3.5 years after the development of distant metastases [5,6].

For patients with slow tumor growth and low burden, active surveillance may be appropriate, with local interventions considered when progression is limited. In contrast, systemic therapy is warranted for rapid and/or symptomatic progression involving multiple lesions or organs. Multikinase inhibitors (MKIs) such as lenvatinib and sorafenib have been approved as first-line treatments for advanced, progressive RAI-R DTC by both the European Medicines Agency (EMA) and the United States Food and Drug Administration (FDA), based on the SELECT and DECISION phase III trials [7,8]. More recently, cabozantinib has emerged as a second-line option for patients who have progress on initial MKI therapy targeting the vascular endothelial growth factor (VEGF) receptor (COSMIC-311 trial) [9,10]. These targeted therapies have demonstrated efficacy in delaying disease progression and, in some cases, improving OS.

Advances in next-generation sequencing (NGS) have enabled the identification of genetic alterations in TC, significantly impacting patient management. Novel selective targeted therapies—including rearranged during transfection (RET) inhibitors (selpercatinib and pralsetinib) and neurotrophic tyrosine receptor kinase (NTRK) inhibitors (larotrectinib and entrectinib)—have further expanded the therapeutic options for selected RAI-R TC cases [11,12,13,14]. Additionally, immune checkpoint inhibitors (ICIs) have shown promising results and are still under investigation [15,16,17].

The optimal timing for initiating MKIs in patients with RAI-R DTC remains controversial. Although MKIs extend progression-free survival (PFS), they are not curative and are associated with significant toxicities that can negatively impact health-related quality of life (HRQoL) [7,18,19]. Consequently, close monitoring of disease activity is essential to determine the appropriate moment for systemic intervention.

Given the lack of a robust consensus on the optimal timing for MKI initiation, particularly in asymptomatic progressive disease, and the emergence of novel targeted therapies, this narrative review aims to synthesize current evidence and best practices to guide clinical decision-making in the management of RAI-R DTC.

## 2. Pathophysiology of RAI-R TC

Several genetic mutations, chromosomal rearrangements, and molecular alterations in various signaling pathways reduce iodine uptake in tumor cells, contributing to refractoriness in DTC. The sodium/iodide symporter (NIS), located on the basolateral membrane of thyroid follicular cells, is essential for actively transporting iodine necessary for synthesizing thyroxine (T4) and triiodothyronine (T3). This process also involves crucial molecules, including the pendrin transporter and thyroid peroxidase (TPO). TPO catalyzes the iodination of thyroglobulin (TG) to form monoiodotyrosine (MIT) and diiodotyrosine (DIT) and subsequently mediates the coupling of these iodinated tyrosines to produce T3 and T4 [20]. Alterations in the expression or subcellular localization of these proteins, particularly NIS, are considered the primary mechanisms underlying radioiodine refractoriness in DTC [21].

### 2.1. Alteration of Signaling Pathways

The Mitogen-activated protein kinase (MAPK) and phosphoinositide 3-kinase/protein kinase Bα/mammalian target of rapamycin (PI3K/AKT/mTOR) pathways are central to TC development and progression, with mutations in genes such as BRAF, RAS, and PTEN playing key roles in RAI-R disease.

- MAPK pathway:

This pathway plays a central role in thyroid cell differentiation and proliferation.

Its dysregulation triggers dedifferentiation in DTC, characterized by reduced expression of genes essential for thyroid hormone biosynthesis, including NIS, TPO, and TG. A pivotal event is decreased histone acetylation at the NIS gene promoter, which diminishes iodine uptake [22].

Chronic activation of both the MAPK and PI3K pathways leads to uncontrolled cell proliferation, impaired apoptosis, enhanced dedifferentiation, increased migration, and angiogenesis [23].

Abnormal MAPK activation in RAI-R DTC is most commonly driven by the BRAF^V600E^ mutation; however, other BRAF mutations, rearrangements, RAS mutations, and MEK gene alterations also contribute [24,25].

- PI3K/AKT pathway:

The PI3K pathway consists of three key molecules: PI3K, AKT, and mTOR. Its activation, alongside cAMP-independent signaling, suppresses the cAMP-dependent pathway responsible for thyroid-specific protein expression, including NIS [26]. Mutations in PIK3CA and alterations in PTEN further drive TC progression and RAI resistance [26]. Overactivation of this pathway is believed to enhance tumor cell survival and proliferation.

- Transforming growth factor-β (TGF-β)/Smad pathway:

Abnormal TGF-β signaling, acting as a tumor promoter, is implicated in TC. In papillary TC (PTC), the BRAF^V600E^ mutation upregulates functional TGF-β1, establishing an autocrine loop that increases Smad3 phosphorylation and levels. This, in turn, enhances NADPH oxidase 4 (NOX4) expression via a complex with p22phox, producing reactive oxygen species (ROS) that serve as second messengers to inhibit differentiation and promote proliferation and metastasis [27]. Thus, the TGF-β/Smad pathway is integrated to BRAF^V600E^-induced RAI-R DTC [28].

- Wnt/β-catetin pathway:

The Wnt family of secreted glycoproteins activates β-catenin, a transcription factor critical for cell growth and differentiation. In TC, particularly those harboring BRAF^V600E^ mutations, β-catenin is markedly elevated in cancer stem cells, supporting their self-renewal and contributing to chemotherapy resistance [29]. Abnormal β-catenin activation can also alter NIS localization, further contributing to RAI resistance [30].

- Notch pathway:

The Notch receptor is a versatile transmembrane protein that influences cell differentiation, proliferation, and survival. In dedifferentiated TC, upregulation of Notch receptor expression helps restore differentiation by enhancing thyroid-specific gene expression like NIS and TPO and reducing tumor growth [31].

- Thyroid-stimulating hormone receptor (TSHR) pathway:

Thyroid-stimulating hormone (TSH) binds to its receptor (TSHR), a member of the G protein-coupled receptor (GPCR), and activates signaling cascades that prove vital for thyroid cell growth and hormone synthesis [32]. In addition, the TSH-TSHR axis contributes to immune evasion. TSHα and TSHβ2, abundantly expressed in monocyte-derived dendritic cells (moDCs), upregulate immune checkpoint protein programmed death-ligand 1 (PD-L1) via the TSHR/AC/PKA/JNK/c-JUN pathway, a process that results in the inhibition of T cell-mediated immune responses against TC cells [33].

### 2.2. Tumor Genetic Profiling

- BRAF mutation and rearrangement:

The most prevalent BRAF mutation in TC is BRAF^V600E^, which substitutes valine with glutamic acid at codon 60, leading to constitutive activation of the MAPK pathway [34]. This mutation is present in approximately 40–60% of PTCs and is associated with reduced NIS expression, increased tumor aggressiveness, higher recurrence rates, lymph node metastasis, and RAI refractoriness [34].

BRAF rearrangements, such as the BRAF-KIAA1549 fusion, are less common but can indicate a more aggressive disease course. Efanov et al. [35] studied the PTC tissues of 65 Ukrainian Americans affected by Chernobyl radiation using targeted NGS and RNA sequencing. Their findings revealed rearrangements in MACF-BRAF, MBP-BRAF, and POR-BRAF, which were identified as key mechanisms driving TC associated with radiation exposure from the Chernobyl disaster.

- RAS mutation:

The RAS gene family comprises three key genes: HRAS, KRAS, and NRAS. These genes encode proteins that regulate cell signaling pathways essential for growth and differentiation. Activating mutations in these genes occur in approximately 20–30% of follicular TC (FTC) and 10-15% of PTC, and they appear less frequently in anaplastic TC (ATC). These mutations often disrupt critical downstream signaling pathways, including the MAPK/ERK and PI3K/AKT pathways, which play vital roles in cell proliferation and survival. Moreover, RAS mutations may interact with other genetic changes, such as BRAF mutations in PTC, thereby affecting tumor behavior and treatment responses [36]. Overall, RAS mutations associate with a more aggressive tumor phenotype.

- RET rearrangement:

RET is a proto-oncogene that encodes a receptor tyrosine kinase. Its rearrangement, commonly seen in PTC, especially in RET/PTC1 and RET/PTC3 fusions, results in constitutive activation of RET signaling, promoting tumor growth and survival [37]. RET rearrangements are often associated with childhood radiation exposure, while germline RET mutations are a hallmark of medullary TC (MTC) and familial syndromes like Multiple Endocrine Neoplasia type 2 (MEN2).

- Telomerase reverse transcriptase (TERT) promoter mutation:

Notably, the coexistence of BRAF^V600E^ and TERT promoter mutations increases the likelihood of RAI refractoriness due to diminished iodine uptake [38].

The TERT gene is essential for telomere maintenance. Mutations in its promoter region create binding sites for ETS transcription factors, leading to telomerase reactivation and enabling limitless cell division [38]. TERT promoter mutations frequently co-occur with BRAF^V600E^, RAS, or TP53 mutations. Notably, the coexistence of BRAF^V600E^ and TERT promoter mutations increases the likelihood of RAI refractoriness due to diminished iodine uptake [39].

- NTRK fusion:

Fusions involving NTRK1, NTRK2, and NTRK3 result in chimeric proteins with constitutive kinase activity activating downstream signaling pathways such as MAPK and PI3K/AKT [14]. Patients harboring NTRK fusion exhibit more aggressive tumor behavior, with a higher risk of distant metastases and RAI refractoriness compared to those with RAS or BRAF mutations [40].

- Anaplastic lymphoma kinase (ALK) mutation and rearrangement:

Although more commonly associated with lung cancer, ALK gene fusions or rearrangements have been identified in TC, particularly in more aggressive forms such as ATC and occasionally in PTC [41].

- SWI/SNF (SWItch/Sucrose Non-Fermentable) complex mutation:

The SWI/SNF complex, which includes subunits like SMARCA4, SMARCB1, ARID1A, ARID1B, SMARCE1, and PBRM1, regulates chromatin remodeling and gene transcription. Mutations or deletions in these subunits, especially in ATC and poorly DTC, can promote BRAF^V600E^-driven tumorigenesis and can impair the efficacy of MAPK inhibitors and redifferentiation therapies [42].

### 2.3. Tumor Microenvironment

The tumor microenvironment in RAI-R TC is marked by increased angiogenesis and mechanisms of immune evasion. Tumor angiogenesis, primarily driven by VEGF signaling, not only promotes tumor growth but also metastasis, making VEGF a key target in the development of systemic treatment strategies.

Additionally, the combined impact of reduced iodine uptake and genetic alterations underscores the need for systemic therapies that can target these pathways and address both the tumor cells and their supportive microenvironment to effectively halt disease progression.

## 3. Current Systemic Therapeutic Options

Several systemic therapies have been developed for the treatment of RAI-R TC. Among these, tyrosine kinase inhibitors (TKIs) have emerged as the most widely used approach, demonstrating notable improvements in PFS and, in some studies, OS, albeit with significant treatment-related toxicity.

### 3.1. TKIs

TKIs are a class of targeted cancer therapies that competitively inhibit adenosine triphosphate (ATP) binding at the catalytic site of tyrosine kinases and serve as the cornerstone of systemic treatment for RAI-R TC. Landmark phase III clinical trials have led to the approved of lenvatinib, sorafenib, and cabozantinib for differentiated TC, resulting in significant improvements in PFS. Although these agents provide meaningful clinical benefits, their impact on OS remains modest, and their use carries substantial adverse effects.

- Lenvatinib:

Lenvatinib is an oral TKI targeting VEGFR1-3, FGFR1-4, PDGFRβ, RET, and KIT. The SELECT phase III trial demonstrated that lenvatinib significantly prolonged PFS compared to placebo (18.3 months vs. 3.6 months, hazard ratio (HR) for progression or death 0.21, 99% CI 0.14–0.31, *p* < 0.00) in 392 patients with RAI-R DTC, treated in first or second line (with up to one prior VEGF- or VEGFR-targeted therapy) [7]. The objective response rate (ORR) was 65% and the median OS had not been reached. Furthermore, mature data revealed that lenvatinib responders experienced a markedly prolonged OS compared to non-responders (52.2 vs. 19.0 months, HR 0.32, 95% CI 0.23–0.46), and patients with a low (≤40 mm) baseline tumor burden had significantly longer OS than those with a high (>40 mm) tumor burden (median OS not reached vs. 29.1 months, HR 0.42, 95% CI 0.28–0.63) [43]. The most common any-grade treatment-related adverse events (AEs) (all grades) were hypertension (68%), diarrhea (59%), decreased appetite (50%), weight loss (46%), and nausea (41%). Of these common events, a subset were severe (grade 3–4): hypertension (41.8%), diarrhea (8%), decreased appetite (5.4%), weight loss (9.6%), and nausea (2.3%).

Dose reductions occurred in 78.5% of patients, and treatment discontinuation due to AEs was required in 14.2% of patients [44].

- Sorafenib:

Sorafenib is an oral inhibitor of VEGFR-1, PDGFRβ, Raf kinases, BRAF, c-KIT, FLT-3, and CRAF. The DECISION phase III trial demonstrated that sorafenib significantly improved PFS compared to placebo (10.8 months vs. 5.8 months, HR 0.58, 95% CI 0.45–0.75, *p* < 0.0001) in 417 patients with RAI-R DTC [8]. The ORR was 12%, and stable disease for at least 6 months was achieved in 42%. The median OS did not differ significantly between groups. The most common any-grade treatment-related AEs included palmar–plantar erythrodysesthesia (76%), diarrhea (69%), alopecia (67%), rash/desquamation (50%), fatigue (50%), weight loss (47%), and hypertension (41%). Among these common events, only a subset were grade 3–4: palmar–plantar erythrodysesthesia (20%), diarrhea (6%), rash/desquamation (5%), fatigue (6%), weight loss (6%), and hypertension (10%). Dose reductions occurred in 66.2% of patients and treatment discontinuation due to AEs was required in 18.8% of patients [43].

- Cabozantinib:

Cabozantinib is an oral TKI that primarily targets MET, VEGFR2, RET, c-KIT, FLT-3, and AXL [45,46]. In the phase III COSMIC-311 trial, cabozantinib demonstrated an improvement in PFS compared to placebo (median not reached vs. 1.9 months; *p* < 0.0001) in 258 patients with RAI-R DTC who had received prior treatment [9]. The ORR was 11%. In the mature data, despite 40 patients in the placebo group crossing over to cabozantinib, a trend toward improved OS was observed in the cabozantinib arm, with an HR of 0.76 (95% CI, 0.45–1.31) [47]. The most common treatment-related AEs of any grade were diarrhea (62%), palmar–plantar erythrodysesthesia (47%), and hypertension (32%). Of these, grade 3-4 were as follows: diarrhea (8%), palmar–plantar erythrodysesthesia (10%), and hypertension (11%) [46,48].

In addition, phase III trials in MTC demonstrated a significantly PFS benefit compared to placebo (11.2 vs. 4.0 months) [45,49].

- Vandetanib

Vandetanib is a TKI that targets RET, VEGFR, and EGFR, approved by the FDA in 2011 and EMA in 2012 for treating advanced MTC. Clinical trials have shown that vandetanib significantly improves PFS in MTC patients [50,51]. However, its impact on OS remains inconclusive [52]. Vandetanib is generally well tolerated, but can cause AEs such as fatigue, hypertension, QTc prolongation, and diarrhea [53,54]. The drug’s efficacy may be limited by the development of resistance over time [55]. Ongoing research is exploring vandetanib’s long-term efficacy, potential combinations with other agents, and strategies to overcome resistance [52,55]. Despite its limitations, vandetanib represents a significant advancement in the treatment of advanced MTC.

- TKIs with evidence from phase II clinical trials:

Entrectinib is an oral TKI targeting NTRK, ROS1, and ALK fusions, approved by the FDA and EMA for ROS1-positive non-small cell lung cancer and NTRK fusion-positive solid tumors, including TC, in patients ≥ 12 years [56,57,58]. It has shown efficacy in NTRK-rearranged TC, which typically exhibits aggressive clinical behavior and a high metastatic risk [59]. In a case report, entrectinib demonstrated a response in a patient with metastatic PTC harboring an EZR-ROS1 fusion [60]. Clinical trials have reported durable responses and intracranial activity [61], with a favorable safety profile marked primarily by low-grade AEs [60]. Entrectinib offers a promising option for NTRK fusion-positive TC, for which no approved drugs were previously available [62,63].

Larotrectinib is a selective inhibitor of tropomyosin receptor kinases (TRKs), specifically TRKA, TRKB, and TRKC, encoded by NTRK1-3. Its mechanism of action involves blocking downstream signaling pathways such as RAS/MAPK, PI3K/AKT, and PLCγ, thereby inhibiting tumor proliferation, survival, and migration.

Approved by both the FDA and EMA, larotrectinib has reported high ORRs (71% to 90%) in DTC patients [64], with rapid and durable responses [65], and a favorable safety profile, with mostly grade 1–2 AEs [66]. The treatment has shown potential for improved long-term outcomes compared to standard care in metastatic DTC [67]. Interestingly, larotrectinib may exert a redifferentiating effect in some RAI-R TC, potentially restoring iodine uptake [68]. These findings support routine testing for NTRK gene fusions in advanced non-MTC patients before initiating systemic therapy [64].

Axitinib, a selective inhibitor of VEGFRs, demonstrated antitumor activity across all histologic TC subtypes in phase II trials, with ORRs of 30–38% and median PFS of 15–18.1 months [69]. A compassionate use program in Spain reported superior outcomes with first-line axitinib compared to its use in second-line settings [70].

Pharmacokinetic/pharmacodynamic analyses further suggest that higher axitinib plasma levels correlate with greater tumor size reduction [69].

The efficacy of sunitinib is attributed to its multi-targeted inhibition of VEGFR, PDGF-R, RET, c-KIT, FLT3, and receptor of macrophage colony-stimulating factor (CSF1R) [71]. Clinical studies have reported partial response rates ranging from 13% to 55.5% in DTC and 0% to 55% in MTC [71,72]. Notably, sunitinib is associated with a high incidence of hypothyroidism, possibly due to its inhibition effect on TPO activity [73].

Pazopanib, an MKI targeting VEGFR and PDGFR, has shown promising activity in DTC, with a 49% ORR [74].

Mechanisms of action for these drugs are summarized in Table 1.

### 3.2. Immune Checkpoint Inhibitors (ICIs)

The role of immunotherapy in RAI-R TC is still evolving, with several ongoing clinical trials investigating its potential. ICIs, such as pembrolizumab (anti-PD-1), may offer benefit in advanced TC, particularly when combined with TKIs.

- Pembrolizumab:

In advanced DTC, pembrolizumab monotherapy has shown limited efficacy, with an ORR of 9% [82]. In ATC, pembrolizumab combined with chemoradiotherapy as initial treatment yielded disappointing survival outcomes [83]. However, adding pembrolizumab to kinase inhibitors at disease progression has shown potential benefit in some ATC patients [84,85]. Notably, the combination of lenvatinib and pembrolizumab demonstrated promising results in ATC and poorly DTC, with a median PFS of 17.75 months and some long-term remissions [17]. In patients with RAI-R DTC progressing on lenvatinib, the addition of pembrolizumab resulted in a 15% partial response rate and a median PFS of 12.6 months [86]. These findings suggest that pembrolizumab, particularly in combination regimens, may have a role in advanced TC treatment. 

### 3.3. Chemotherapy

Traditional chemotherapy has limited efficacy in TC and is generally reserved for patients who have exhausted other treatment options. Agents such as doxorubicin were used historically, but their role in current treatment protocols is minimal due to the advent of more effective targeted therapies.

### 3.4. Other Novel Therapies

- RET Inhibitors:

Targeted therapies against RET fusions, such as selpercatinib (LOXO-292) and pralsetinib (BLU-667), have shown remarkable efficacy in RET-altered TCs.

The LIBRETTO-001 trial demonstrated durable responses in RET fusion-positive TC, with ORRs of 95.8% in treatment-naïve patients and 85.4% in pre-treated patients [87]. In RET-mutant MTC, ORRs were 81.0% in patients naïve to cabozantinib/vandetanib and 73.5% in pre-treated patients [88]. Selpercatinib exhibited a consistent safety profile, with common AEs including hypertension and increased liver enzymes [89]. Based on these results, selpercatinib has received FDA and EMA approval for treating RET-altered TC and lung cancers [90,91].

Similarly, pralsetinib has shown high ORRs (73.1–90.9%) and durable responses, with median PFS of 25.4–25.8 months across various cohorts in the ARROW study [81,92]. Consequently, the FDA granted accelerated approval for pralsetinib for TC harboring RET mutations or fusions, although its label included warnings for AEs such as pneumonitis, hypertension, and hepatotoxicity [93].

- BRAF Inhibitors:

In patients with BRAF^V600E^-mutant RAI-R TC, targeted therapy with the BRAF inhibitor dabrafenib in combination with the MEK inhibitor trametinib has demonstrated promising efficacy, especially in ATC. Phase II trials have reported high ORR ranging from 56% to 73.1% in both ATC and PTC [94,95,96]. This combination has been associated with improved long-term survival in ATC patients, with 12-month OS rates reported between 51.7% and 80% [94,95]. Real-world data support these findings, showing median OS of 6.9 months in ATC patients [97]. Additionally, the combination therapy may induce redifferentiation in poorly DTC [98]. However, dose reductions due to AEs are common, and some studies suggest that the combination may not offer significant advantages over dabrafenib monotherapy in RAI-R TC [96,99].

Neoadjuvant BRAF-directed therapy followed by surgery has also been explored in stage IVb ATC, showing improved survival (12-month OS of 93.6% and PFS of 84.4%), reduced tumor size, and lower surgical morbidity, though outcomes were poorer in patients with residual disease post-surgery; these promising findings require validation in prospective trials [100].

Preclinical studies have further highlighted the potential of combining targeted agents. Trametinib, alone or with pazopanib, effectively inhibited TC growth in cell lines and xenograft models [101]. A phase I trial of pazopanib and trametinib in DTC showed a 33% ORR and median PFS of 10.7 months [102]. Intermittent dosing of dabrafenib and trametinib in BRAF^V600E^-mutated PTC also produced encouraging responses in case reports [103]. Although pazopanib monotherapy showed a 14.3% partial response rate and a 9.4-month median PFS in DTC [104], its single-agent activity in ATC remains limited despite preclinical promise [74]. Adding pembrolizumab to these regimens may further enhance survival outcomes [105].

Together, these studies underscore the potential of novel targeted therapies in various TC subtypes, warranting further investigation in prospective clinical trials.

## 4. Clinical Indications for Systemic Therapy

Systemic therapy should be initiated in patients with progressive or symptomatic RAI-R TC, as well as those with high-risk metastatic disease. Current guidelines from the American Thyroid Association (ATA) and European Society for Medical Oncology (ESMO) offer clear criteria for patient selection.

### 4.1. Criteria for Determining Refractoriness (RAI-R) [106]

- Tumors that show no RAI uptake in all local or distant lesions on a post-treatment whole-body scan, despite being DTC.
- Partial loss of RAI uptake, with some lesions failing to concentrate RAI.
- Progressive disease within 6–12 months, despite receiving adequate doses of RAI, as defined by RECIST criteria.
- Patients who have received a cumulative RAI dose (typically >600 mCi) without a significant response.
- Locally advanced thyroid tumors in which surgical resection is not feasible, precluding proper assessment of RAI uptake.

### 4.2. Criteria for Determining Advanced/Aggressive Forms

According to the framework developed by the American Head and Neck Society (AHNS) and the International Thyroid Oncology Group, advanced TC is defined by several dimensions [107]:

- Structural/surgical category:
  - Invasive or inoperable locoregional disease.
  - Recurrence of the disease.
  - Presence of distant metastases.
  - Rapid progression detectable on imaging studies.
- Biochemical category:
  - Tumors resistant to RAI treatment.
  - Tumors unresponsive to TSH suppression therapy.
  - Rapid doubling time of specific biomarkers (e.g., TG).
- Histologic/molecular category:
  - Aggressive histologic variants (e.g., poorly DTC or ATC).
  - High Ki67 proliferation index.
  - Elevated mitotic count.
- Clinical judgment:

Physicians may also designate cases as aggressive based on observed clinical behavior, even if all criteria are not met.

It is important to note that a recent consortium of experts from the ATA, ETA, the European Association of Nuclear Medicine, and the Society of Nuclear Medicine and Molecular Imaging has highlighted that no single definition, classification, criterion, or clinical scenario serves as an absolute indicator of RAI-R DTC [108].

### 4.3. Patient Selection

In RAI-R TC, the decision to initiate systemic therapy should be made by specialized endocrinologists or oncologists, considering several factors, such as disease progression, tumor burden, symptoms, and patient-specific considerations [109].

Molecular testing has become an invaluable tool in the diagnosis and management of TC. Advanced techniques, like targeted NGS panels (e.g., ThyroSeq), can accurately detect mutations in multiple cancer-related genes with high accuracy and sensitivity, while requiring minimal DNA input [110]. This is particularly useful in cases with indeterminate cytology, where the presence of mutations correlates with a higher risk of malignancy [110]. By complementing fine-needle aspiration cytology, molecular testing enhances diagnostic accuracy and guides clinical management [111,112]. Additionally, molecular profiling is essential for characterizing advanced TC and developing targeted therapies [107].

Genomic testing should be considered for patients with advanced or metastatic RAI-R DTC under specific conditions: (a) the patient has good performance status, (b) systemic treatment is planned, (c) first-line MKI therapy has failed, and (d) access to targeted therapies is feasible [107]. Molecular analysis may be performed at various stages of RAI-R disease progression, at diagnosis, if possible, upon structural progression (as defined by RECIST criteria), or after progression following one or two lines of MKI treatment [109].

Further research is needed to optimize treatment sequencing and address potential drug resistance [113].

## 5. Timing of Systemic Therapy

Various guidelines recommend systemic therapies for patients with progressive, symptomatic, or metastatic RAI-R TC, and address the timing for starting MKIs. For instance, NCCN guidelines recommend considering MKIs for patients with rapidly growing or symptomatic lesions [114]. The ATA guidelines suggest MKI administration for patients with life-threatening lesions, diffuse disease progression, or symptomatic disease [115], while ESMO guidelines advise their use in patients with multiple symptomatic lesions or asymptomatic patients with progressive multifocal disease [116]. The ETA emphasizes the importance of patient-related factors (age, health status, comorbidities, and contraindications) and patient preferences [117]. Similarly, the Japan Association of Endocrine Surgeons’ guidelines indicate that MKIs should not be started unconditionally in all patients with rapid tumor growth or symptoms; instead, the timing should balance potential benefits against risks in the context of the patient’s overall condition [118]. Indeed, patients exhibiting asymptomatic, stable disease can be closely monitored without immediate intervention, especially in cases of low tumor burden [118]. The optimal sequence of MKIs remains undetermined due to limited evidence from subgroup analyses of phase III trials.

**Figure 1:**
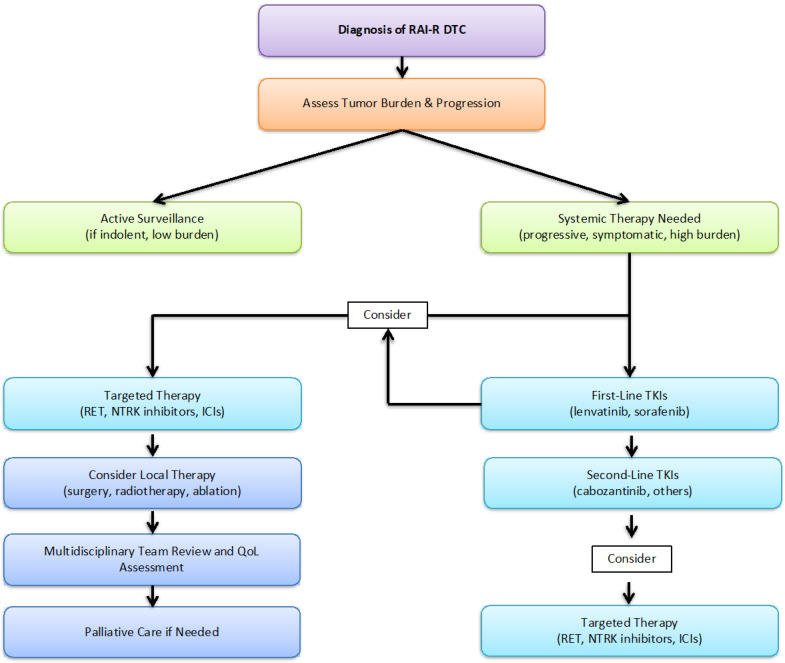
Therapeutic sequencing and decision-making pathway for RAI-R DTC.

The decision to initiate systemic therapy is critical. While early intervention may prevent disease complications, it must be balanced against the risks of long-term toxicity, particularly in patients with slow-growing tumors (Figure 1, Table 2).

### 5.1. Early vs. Late Intervention

- Early intervention:

Some experts advocate for the early initiation of systemic therapy in metastatic disease to prevent complications such as bone fractures or organ failure. However, this approach may expose patients to treatment-related side effects for an extended period [7,8].

- Late intervention:

Delaying systemic therapy until symptomatic progression is another approach, particularly for patients with indolent disease. This allows patients to avoid toxicities until the disease becomes more aggressive [115,119].

In a global non-interventional study by Brose et al. [120], the median time to symptomatic progression in asymptomatic RAI-R DTC patients treated with MKIs was 55.4 months overall, 55.4 months in the cohort that initiated MKI treatment at study entry, and 51.4 months in the cohort that did not initiate MKI treatment at study entry. Median PFS from the start of MKI therapy was 19.2 months and from the start of sorafenib therapy 16.7 months, while median OS from RAI-R classification was 167 months. For sorafenib-treated patients, 70% required dose modifications, and 89% experienced treatment-emergent AEs. The study provides valuable real-world insights into outcomes for patients with asymptomatic, progressive RAI-R DTC under observation or MKI treatment in the current era.

### 5.2. Active Surveillance

Although some patients are refractory to RAI therapy, they may maintain stable metastatic disease for several years without additional treatment [121]. The therapeutic approach is primarily guided by tumor burden and the rate of disease progression. For lesions < 1–2 cm, active surveillance is generally recommended [122]. Studies have shown that small metastatic lymph nodes (<1 cm) and thyroid bed nodules can be safely monitored over long periods using ultrasound [123,124,125]. Similarly, small soft tissue metastases can be followed with cross-sectional imaging. Depending on their location, stable or very slowly progressing lesions (i.e., without significant growth over 12–16 months) may also be managed conservatively, except when they are near critical structures, where even minimal growth could cause symptoms [126].

### 5.3. Integration with Local Treatments

- Surgery

For patients with oligometastatic disease (≤5 metastatic lesions), surgical resection may be effectively combined with systemic therapy. In DTC patients with extracervical metastases, a multifaceted approach is essential for accurate diagnosis, treatment, and monitoring. While the initial goal is cure, especially for small, radioiodine-avid pulmonary metastases, the focus shifts to improving survival and managing symptoms in persistent disease. Asymptomatic, stable RAI-R metastases may be managed with active surveillance, whereas large or painful lesions often require directed therapies such as external beam radiotherapy (EBRT), surgery, or embolization [127]. Multimodal imaging (CT, MRI, bone scans, and ^18^FDG-PET) plays a key role in guiding treatment decisions.

- Radiation therapy

EBRT is an important adjunct in the management of TC, particularly for high-risk patients with bone or brain metastases, when used in conjunction with systemic therapies. In DTC, EBRT can improve local control in patients over 45 with microscopic residual disease or extensive extrathyroid invasion [128]. It is also recommended for gross residual or unresectable disease [129]. In MTC, EBRT may benefit patients with surgically inaccessible or residual tumors [130]. For ATC, a combination of surgery, EBRT, and chemotherapy can yield higher local control rates and prolonged survival [131]. Advances in EBRT techniques, such as intensity-modulated radiation therapy, can improve treatment accuracy and reduce toxicity [132]. However, the impact of EBRT on OS remains uncertain, and its routine use is still debated in the absence of randomized controlled studies [133,134].

## 6. AEs and Toxicity Management

Systemic therapies, especially TKIs, have improved outcomes in advanced TC, but are associated with numerous AEs, particularly during the initial phases of therapy. Common AEs affect the cardiovascular, gastrointestinal, dermatological, and metabolic systems, with over 90% of patients experiencing at least one AE [135]. Hypothyroidism is the most frequently observed endocrine toxicity [136]. Furthermore, TKIs significantly increase the risk of arterial thromboembolic events, although venous events are not similarly affected [137]. Early assessment and management of AEs are crucial to mitigate their impact on QoL and to avoid treatment discontinuation [138].

In a prospective real-world study involving 20 patients treated with lenvatinib, 100% experienced at least one AE, and 80% had grade ≥ 3 toxicity. The most commonly self-reported symptoms included fatigue, anorexia, abdominal pain, xerostomia, and gastrointestinal disturbances, all of which significantly interfered with daily activities. HRQoL, assessed using the EQ-5D-3L and EQ-VAS questionnaires, showed a statistically significant decline at 3 and 6 months of treatment (*p* < 0.05), followed by partial recovery at 12 months, possibly due to dose adjustments and proactive symptom management [139]. These findings were complemented by the phase II randomized, double-blind Study 211, which compared HRQoL in patients with RAI-R DTC initiating lenvatinib at either 18 mg/day or the approved 24 mg/day starting dose. No statistically significant differences were found between the two arms in longitudinal changes in EQ-5D-3L or FACT-G scores. Similarly, time to deterioration of HRQoL was comparable (16–28 weeks), and no clinical benefit was observed with the lower starting dose [140]. Both studies identified a trend toward better HRQoL preservation in patients who achieved objective tumor responses, suggesting that disease control may positively influence overall well-being.

Despite the high frequency of AEs, QoL can be maintained—or even restored—through appropriate toxicity management and close monitoring. The integration of patient-reported outcome measures allows clinicians to detect symptoms that might be overlooked during routine assessments and supports a more patient-centered approach to care. A multidisciplinary approach, including dedicated endocrinologists, is recommended for optimal management of TKI-related AEs [141,142]. Regular monitoring of thyroid function is essential throughout treatment [143]. In addition, prehabilitation measures prior to initiating TKIs—particularly lenvatinib—may enhance patient resilience and reduce toxicity. Such measures include optimizing fluid and electrolyte balance, adjusting dietary intake, and implementing appropriate skin care protocols. For patients with pre-existing hypertension, intensifying antihypertensive treatment and optimizing blood pressure control are also recommended [144]. These strategies aim to minimize the severity of AEs and support better treatment tolerance and overall outcomes.

### 6.1. Common AEs

Over 90% of patients receiving TKIs experience at least one AE, with a higher incidence in the first 6–8 weeks of treatment. Frequently affected systems include dermatological, gastrointestinal, cardiovascular, and metabolic, potentially compromising patient HRQoL. For lenvatinib, the most common AEs are hypertension (67%), fatigue (66%), diarrhea (59%), and decreased appetite (50%) [7]. Serious events, such as proteinuria and thromboembolic events, occur in a minority of patients. Common AEs from sorafenib include hand–foot syndrome (76%), rash (50%), and alopecia (67%) [44].

In a study of 29 patients on MKIs, 17.2% experienced rare AEs [145]. These included heart failure, thrombocytopenia, and squamous cell carcinoma during sorafenib treatment, as well as squamous cell carcinoma and oophoritis with intestinal perforation during vandetanib therapy. The study followed patients for a mean of 13.7 months and included individuals with DTC and MTC. While most MKI-related adverse effects are manageable, the authors emphasize the importance of being aware of these rare complications.

### 6.2. Toxicity Management

Dose reductions or temporary treatment discontinuation may be necessary in cases of severe toxicities, particularly in older or frail patients.

Management strategies for common AEs include the following:

- Fatigue/asthenia

Thyroid function should be assessed before starting lenvatinib and monitored monthly, with adjustments to thyroid-replacement therapy as needed. If no other cause is found, supportive care, such as proper nutrition, exercise, and stress reduction techniques, is recommended. For moderate to severe fatigue, consider temporary dose interruptions of MKIs.

- Hypertension

MKI-induced hypertension is thought to result from decreased nitric oxide production resulting from VEGF inhibition. Hypertension caused by antiangiogenic therapies is believed to reflect the agent’s intended pharmacologic activity. This AE, which may correlate with prolonged PFS, requires proactive management with antihypertensive medications to mitigate risks [146].

- Proteinuria

Before initiating MKIs, baseline urinalysis and protein-to-creatinine ratio assessments are recommended. Decisions regarding dose modification or treatment interruption should be individualized, as proteinuria is a class effect, and switching agents may not be beneficial.

- Gastrointestinal AEs

Management includes dietary modifications (avoiding foods that worsen diarrhea and promoting stool consistency), dehydration control, use of loperamide, and dose adjustments for severe AEs (grades 3 or 4). Once symptoms subside, optimal dosing may be resumed.

- Stomatitis/mucositis

Patients should avoid irritants such as mint-flavored toothpaste, alcohol-based mouthwashes, and spicy or acidic foods. Ongoing clinical trials are evaluating steroid mouthwashes, which may reduce the incidence and severity of stomatitis in patients on lenvatinib. Topical treatment (lidocaine or steroid ointment) can provide relief. For severe stomatitis (grade ≥ 3), dose modifications are recommended [147].

- Palmar–plantar erythrodysesthesia syndrome (PPES):

Patients should be advised to minimize exposure of hands and feet to hot water, avoid tight footwear, and limit vigorous exercise to prevent PPES. This condition typically develops within the first 2–4 weeks of TKI therapy, so avoiding trauma and ensuring adequate rest during this period is crucial. Dose reduction or interruption may be required until symptoms return to grade ≤ 1 [148].

- Hemorrhagic events

Mechanisms may include blood vessel destabilization, vascular integrity loss, and thrombocytopenia. MKIs should be withheld in patients with grade 3 hemorrhage until resolution to grade 0 or 1. Treatment can resume at a reduced dose, depending on the event’s severity. For grade 4 hemorrhages, MKI should be permanently discontinued [146].

- Hepatotoxicity

TKIs can induce liver injury through mitochondrial toxicity, inhibition of glycolysis, and formation of reactive metabolites, ranging from mild elevation of liver enzymes to severe liver damage [149,150]. Management strategies include close monitoring of liver function tests, dose adjustments, and, in some cases, corticosteroid use or discontinuation of the drug are essential.

Early risk assessment and prompt AE identification are crucial for optimal patient care. A multidisciplinary team, including nursing support, plays a vital role in preventing and managing these side effects to maximize treatment benefits and minimize risks. Effective AE management helps avoid drug discontinuation or interruption that could potentially accelerate disease progression. Based on the tumor’s molecular characteristics and local regulatory framework, a clinician may consider switching to second-line treatment, such as cabozantinib or newer targeted therapies, when necessary.

By supporting patients throughout their clinical-therapeutic journey, healthcare professionals can improve treatment outcomes and enhance patients’ QoL.

## 7. Challenges, Practical Implications, and Future Directions

### 7.1. Challenges and Limitations of Emerging Therapies

Despite the promising results of ICIs and novel targeted therapies in RAI-R TC, several challenges remain. ICIs, particularly pembrolizumab, have shown limited monotherapy efficacy in DTC, and their clinical benefit often requires combination regimens, which can increase toxicity and complicate management [17,82,83,84,85,86]. Moreover, access to ICIs and next-generation targeted therapies is uneven globally, largely due to high costs and the need for specialized molecular testing to identify actionable mutations (e.g., RET and NTRK fusions). Even in high-income countries, financial toxicity can be substantial, impacting both health care systems and individual patients.

Furthermore, many emerging agents (e.g., RET inhibitors, NTRK inhibitors, and novel BRAF/MEK combinations) have primarily been evaluated in small, selected patient populations or in early-phase trials, limiting the generalizability of the results. Long-term data on survival benefit, QoL, and resistance patterns are still lacking. These limitations underscore the need for continued research and real-world studies to better define the optimal use and sequencing of these therapies.

### 7.2. Practical Recommendations for Clinicians and Researchers

To maximize the clinical impact of current evidence, we suggest several actionable steps:

- Routine molecular profiling: All patients with progressive RAI-R TC should undergo comprehensive molecular testing early in the disease course to identify potential targets for therapy.
- Multidisciplinary evaluation: Treatment decisions should involve tumor boards including endocrinologists, oncologists, nuclear medicine specialists, and molecular pathologists to ensure individualized care.
- Cost-effectiveness analysis: Institutions should prioritize the implementation of cost-effectiveness assessments when introducing new targeted therapies, especially in resource-constrained settings.
- Proactive toxicity management: Early recognition and intervention for treatment-related AEs can prolong therapy duration and maintain QoL.
- Participation in clinical trials: Clinicians should encourage eligible patients to enroll in ongoing studies evaluating novel agents or treatment sequences to help build robust evidence.

### 7.3. Integration of Artificial Intelligence (AI) in Clinical Practice

The integration of AI and machine learning technologies offers exciting opportunities to enhance clinical decision-making in RAI-R TC. AI-based algorithms can assist with the following:

- Molecular profiling: AI tools can rapidly analyze NGS data, identifying actionable mutations and predicting potential resistance mechanisms.
- Treatment selection: Predictive models based on real-world data could support clinicians in selecting the most appropriate systemic therapy based on patient and tumor characteristics.
- Monitoring and toxicity prediction: AI-driven digital platforms, including mobile health apps and wearable devices, can enable real-time monitoring of symptoms and early detection of AEs.
- Clinical trial matching: Automated systems can help match patients to appropriate clinical trials based on their molecular profiles and clinical parameters, facilitating access to emerging therapies.

The implementation of AI must be accompanied by rigorous validation, transparency, and careful integration into clinical workflows to ensure its effectiveness and acceptance among healthcare providers.

## 8. Conclusions

The management of RAI-R DTC remains a complex clinical challenge that requires balancing tumor progression, treatment-related toxicity, and HRQoL. The introduction of MKIs such as lenvatinib and sorafenib has significantly improved outcomes in advanced cases, although their AEs necessitate careful monitoring and dose adjustments. At the same time, emerging targeted therapies, including RET and NTRK inhibitors, are expanding treatment options, particularly for patients with actionable genetic alterations, highlighting the potential of precision medicine.

To optimize patient outcomes, clinicians should prioritize molecular profiling at diagnosis or upon disease progression to identify actionable mutations. Early and proactive management of AEs, particularly hypertension, fatigue, and gastrointestinal toxicity, is essential to minimize treatment discontinuation. In selected cases, particularly those with low tumor burden and indolent progression, active surveillance may be considered to delay systemic therapy while maintaining disease control.

Recent advances and challenges in the field—including the accessibility and cost implications of novel therapies, the need for practical guidance for clinicians, and the integration of AI into molecular profiling and treatment selection—offer new opportunities for improving patient care. Addressing these aspects is essential to further personalize therapy, enhance decision-making, and overcome current limitations.

From a research perspective, ongoing clinical trials should focus on defining the optimal sequencing of systemic therapies, addressing drug resistance mechanisms, and improving the risk–benefit ratio of treatment strategies. Future efforts should also explore how digital innovations can support clinical practice, ultimately enhancing QoL and outcomes for patients with RAI-R DTC.

## Figures and Tables

**Table 1:** Available agents in the treatment of radioiodine-refractory differentiated thyroid carcinoma (RAI-R DTC), medullary TC (MTC), and anaplastic TC (ATC).

Drug	Clinical Trial	Phase	Indication	Main Molecular Targets	Dosage	PFS Compared to Placebo (Months)
Lenvatinib [7]	NCT01321554	III	First/second-line DTC	VEGFR1-3, FGFR1-4, PDGFRα, RET, c-KIT	24 mg orally per day	18.3 vs. 3.6, HR 0.21 (99% CI 0.14–0.31, *p* < 0.001)
Sorafenib [8]	NCT00984282	III	First * line DTC	VEGFR1-3, PDGFRβ, Raf, c-KIT, FLT-3. BRAF, CRAF	400 mg orally twice daily	10.8 vs. 5.8, HR 0.58 (95% CI 0.45–0.75, *p* < 0.0001)
Cabozantinib [9,45]	NCT01811212/NCT00704730	III (DTC)/ III (MTC)	Second-line DTC First-line non-RETm MTC Second-line RETm MTC	MET, VEGFR2, RET, c-KIT, FLT-3, AXL	60 mg orally per day	DTC: NE vs. 1.9, HR 0.22 (96% CI 0.13–0.36; *p* < 0.0001) MTC: 11.2 vs. 4.0; HR 0.28 (95% CI 0.19–0.40; *p* < 0.001)
Vandetanib [75]	NCT00537095/NCT00410761	II/III	First-line non-RETm MTC Second-line RETm MTC Subsequent in DTC	VEGFR2/3, EGFR, RET	300 mg orally per day	DTC: 11.1 (95% CI, 7.7–14.0) MTC: 30.5 (model prediction; NE) vs. 19.3, HR 0.46 (95% CI 0.31–0.69; *p* < 0.001)
Entrectinib [76]	NCT02568267	II	Any line	NTRK, ROS1, and ALK fusions	600 mg orally per day	19.9 (95% CI, 6.5–33.8)
Larotrectinib [77]	NCT02122913/ NCT02637687/NCT02576431	I/II	Any line	NTRK fusions	100 mg orally twice daily	44.0 (95% CI, 16.6–NE) at a median follow-up of 38.7 months
Axitinib [69]	NCT00094055	II	Subsequent line if no driver mutation	VEGFR1-3	5 mg orally twice daily	15–18.1 (95% CI)
Sunitinib [78]	NCT00381641	II	Subsequent line if no driver mutation	VEGFR1/2, PDGFR, RET, KIT, FLT3, CSF1R	37.5 mg orally per day	12.8 (95% CI, 8.9–NE)
Pazopanib [79]	NCT00625846	II	Subsequent line if no driver mutation	VEGFR, PDGFR	800 mg orally per day	11.4 (95% CI)
Selpercatinib [80]	NCT04211337	III	First/second-line DTC (targeted therapy) First-line RETm MTC First-line RETm ATC	RET	160 mg orally twice daily	NE vs. 16.8, HR 0.28 (95% CI 0.16–0.48; *p* < 0.001)
Pralsetinib [81]	NCT03037385	II	Targeted therapy	RET	400 mg orally per day	25.9, NR, 25.4 (95% CI) **

PFS: progression-free survival; RETm: RET mutation; NE: not estimable. * Can be considered as a subsequent line. ** 25.9 months in patients with RETm MTC who had received prior cabozantinib/vandetanib; NR in patients with treatment-naïve RETm MTC; 25.4 months in patients with previously treated RET fusion-positive TC.

**Table 2:** Therapeutic sequencing pathway for RAI-R DTC.

	Clinical Situation	Recommended Approach
1	Diagnosis of RAI-R DTC	Assess tumor burden and disease progression
2	Low tumor burden, indolent disease	Active surveillance with periodic monitoring
3	Progressive or symptomatic disease	Systemic therapy initiation (TKIs or targeted agents)
4	First-line systemic therapy	Lenvatinib or Sorafenib
5	Disease progression after first-line therapy	Cabozantinib or alternative systemic agents
6	Molecularly targeted therapy	RET/NTRK inhibitors, immune checkpoint inhibitors
7	Consideration of local treatments	Surgery, radiotherapy, or ablative technique
8	Quality of life assessment	Multidisciplinary team review and supportive care
9	Palliative care if necessary	End-of-life care planning and symptom management

RAI-R: radioiodine-refractory; DTC: differentiated thyroid carcinoma; TKIs: tyrosine kinase inhibitors; RET: rearranged during transfection; NTKR: neurotrophic tyrosine receptor kinase.
